# Physician-patient communication in diagnostic examinations: what is the role of the radiologist?

**DOI:** 10.1590/0100-3984.2017.0084

**Published:** 2018

**Authors:** Jessyca Couto Otoni, Marcela Pecora Cohen, Almir Galvão Vieira Bitencourt

**Affiliations:** 1 MD, Radiologist at the A.C.Camargo Cancer Center, São Paulo, SP, Brazil.; 2 PhD, MD, Radiologist at the A.C.Camargo Cancer Center, São Paulo, SP, Brazil.

**Keywords:** Radiology, Physician-patient relations, Diagnostic imaging, Ethics, Radiologia, Relações médico-paciente, Diagnóstico, Ética

## Abstract

Historically, radiology has developed in a way that has increasingly distanced the radiologist from the patient. Currently, diagnostic imaging results are predominantly communicated through written reports. Written communication is not considered sufficient, verbal communication being essential for the performance of the modern radiologist to be considered satisfactory. However, a lack of preparation on the part of the radiologist when communicating the diagnosis, especially when it is not favorable (as is often the case in a cancer hospital), makes that conversation quite challenging. Studies conducted in other countries have demonstrated that there are a variety of opinions on the part of requesting physicians and patients regarding radiologist-patient communication, which can be explained by cultural differences. Although there is no rule regarding the best way to accomplish such communication, there are definitely incorrect ways. To bridge the gap between radiologists and patients and improve radiologist-patient communication, preparation of radiologists during their medical residency is fundamental. Therefore, it is important to address this question in Brazil. The objective of this study was to identify deeper discussions about the topic in the scientific literature. This analysis could help us map those involved and plan strategies to improve the ethical behavior of radiologists toward their patients.

## Abstract (pt)

A radiologia se desenvolveu, historicamente, de uma maneira que afastou cada vez mais o radiologista do paciente. Atualmente, a comunicação do diagnóstico radiológico é realizada predominantemente por laudos escritos. A comunicação escrita, porém, não pode ser considerada suficiente, sendo a comunicação verbal essencial para a boa atuação do radiologista moderno. Entretanto, a falta de preparo do radiologista na informação do diagnóstico, principalmente quando este não é favorável, como acontece frequentemente em um hospital oncológico, constitui um grande problema para esse especialista. Estudos realizados em outros países demonstraram variedade de opiniões dos médicos solicitantes e dos pacientes quanto à comunicação médico-paciente na radiologia, o que pode ser explicado por diferenças culturais. Embora não haja uma regra sobre a melhor maneira de realizar essa comunicação, há certamente maus caminhos. Para que o distanciamento entre radiologistas e pacientes diminua e a comunicação melhore, é fundamental o preparo do radiologista durante a residência médica. Deste modo, é importante levantar essa questão em nosso meio. Este estudo pretende buscar, na literatura científica, discussões mais profundas acerca do tema, pois essa análise pode nos auxiliar no mapeamento dos envolvidos e, futuramente, planejar estratégias de melhora no comportamento ético do radiologista frente ao paciente.

## INTRODUCTION

Since the discovery of X-rays more than 120 years ago (in 1895), medical practice in diagnostic imaging has undergone great transformations, ranging from its technological evolution to the radiologist-patient relationship. When the specialty was first recognized, at the beginning of the nineteenth century, the radiologists themselves performed the examinations, interpreted the results, and communicated their findings directly to the patient. However, since 1922 in the United States and 1957 in Brazil, there have been qualification and licensing requirements for radiology technicians, who became responsible for performing the examinations, leaving to the radiologist only the role of interpreting the images. That change distanced radiologists from the patients, making the former "invisible" to the latter. Thereafter, diagnostic imaging became a legitimate medical specialty and was recognized as such by professionals working in other (clinical and surgical) specialties; the invention of computed tomography, in the 1970s, further distanced radiologists from patients, leaving them in the rearguard of patient care^(1)^.

The high demand for radiological tests, the practicality of the digital era, and the creation of teleradiology, together with the dissemination of diagnostic and imaging services, have further distanced radiologists from their patients^(2)^. However, there are still examinations and protocols in which the radiologist has greater contact with the patient, such as ultrasound, interventional radiology, and mammography. There is an increasing desire on the part of patients to understand their illness and build an equitable relationship with their physician. Consequently, patients ask for a closer relationship with the imaging specialist, as well as for clearer and more direct communication of the results of their examinations^(3)^. Therefore, it is necessary to bridge the gap between radiologists and their patients.

The objective of this study was to examine the role of the radiologist in physician-patient communication of the results of diagnostic tests. To that end, we have reviewed the literature on the topic.

## THE ROLE OF THE RADIOLOGIST

We know that the main role of the radiologist is to interpret the images, to make diagnostic hypotheses, and to communicate impressions about the examinations. However, what is the best way to communicate the diagnosis? At most facilities, diagnoses are currently communicated through written reports directed to requesting physicians, to whom patients have free access. It has been demonstrated that, in 60-90% of cases, radiologists do not know or have not seen the patient in question^(4)^, and that 70% of all medical malpractice suits are attributable to poor physician-patient communication^(5)^. Those aspects make verbal communication between the radiologist and patient essential for the development of the radiologist and for good medical practices.

There are a number of questions to be answered. Should radiologists communicate the findings of the examination and their impressions directly to patients? Should this always be the responsibility of the radiologist? How do patients feel in the presence of a radiologist? Is the radiologist ready and in the right environment to give a patient bad news?

## OPINIONS OF PATIENTS AND PHYSICIANS

According to Tondeur et al.^(6)^, despite the fact that patients have the right to know about their health status, bad news should not be communicated without certain precautions having been taken. The authors found that many patients do not truly want to know the entire truth and that some patients do not want to know anything. In a study conducted at the University of Texas, Schreiber et al.^(7)^ found that 92% of patients wanted the radiologist to tell them if the diagnosis was normal, 87% wanted to be told by the radiologist even if the result was abnormal, and 7% preferred not to receive a diagnosis (normal or abnormal) from the radiologist unless they themselves asked for one. This indicates that most patients prefer to hear the results of their examination from the radiologist and at the time of the procedure, rather than receiving them from the requesting physician at a later time.

Evaluating physician opinions on this communication, another study conducted at the same institution showed that most physicians, including radiologists, agree that radiologists should report the diagnosis to the patient only when requested, informing the requesting physician of the diagnosis and of whether or not the patient has already been informed^(8)^.

Levitsky et al.^(9)^ carried out a study in which questionnaires about conveying the results of imaging examinations to patients who wish to know were sent to physicians, including radiologists, in various regions of the United States. The authors found that, for normal results, 89% of radiologists and 76% of physicians in other specialties felt that patients should be informed of the results of imaging examinations. For examinations showing severe abnormalities, those proportions were 33% and 28%, respectively. That demonstrates the insecurity of radiologists in transmitting serious diagnoses, which is probably related to their lack of preparation to deal with such situations.

In a study conducted in Belgium^(6)^, where the code of medical ethics states that radiologists should not communicate diagnoses to patients, more than 90% of physicians agreed that radiologist should never, or only in specific circumstances, inform patients of the results of examinations. In contrast, 80% of the patients did not agree that radiologists should never communicate the result at the end of the examination, stating that radiologists should give a sense of the diagnosis and that the requesting physician should communicate the full diagnosis. That can be at least partially attributed to the fact that few patients are aware of the code of medical ethics, as evidenced by the fact that it is common for a patient to ask for the result at the end of the examination. The discrepancy between the results of these studies can be explained by the cultural differences between the countries in which they were conducted.

## ARE RADIOLOGISTS PREPARED?

If an abnormality is discovered while the patient is present, as often occurs, radiologists must choose their first words carefully as they prepare to inform the patient of the finding. In many cases, the radiologist cannot propose a treatment strategy, because that is beyond the scope of the specialty. The task is made even more difficult by the lack of specific training of the radiologist in psychology and in the management of sensitive situations, such as announcing bad news. In situations that are even more stressful, such as the discovery of metastases, there is a greater risk of an improper or disastrous approach by the radiologist, which can be detrimental to the patient. In addition, radiologists do not always know what has already been said to the patients about their illness; hence the importance of multidisciplinary teams and of collaboration between radiologists and requesting physicians.

There is no universal approach to delivering bad news, and radiologists must adapt their approach to each individual case. Although there is no one correct way to announce such news, there are certainly incorrect ways, and radiologists should be aware of certain rules. For example, the conversation should not be held in a corridor or in a hurried manner but rather in a calm, cozy environment that ensures the privacy of the patient. In addition, the radiologist should be at the same level as the patient, looking the patient in the eye, with empathy and respect for the wishes of the patient, and ready to listen, thus guaranteeing the rights of the patient^(10)^.

## ATTEMPTS TO STANDARDIZE PRACTICES

Goske et al.^(5)^ developed the RADPED mnemonic checklist to assist radiology residents in physician-patient communication within the field of pediatric radiology, although it can be applied to radiology in general. The components of the RADPED checklist are **R**apport (creating an affinity with the patient); **A**sk (obtaining information from the patient about the illness and the reason for the examination); **D**iscuss (informing the patient of the steps of the procedure); **P**erform (performing the procedure); **E**xamination (using techniques of distraction, such as movies, music, and toys, during the examination); and (again) **D**iscuss (informing the patient of the outcome of the examination). Only at that time (at the end of the process) is it appropriate to communicate the radiological findings to the patient. When the finding is normal, the conversation is easier for the radiologist and brings considerable relief to the patient. When additional time is needed in order to analyze the images, the radiologist should inform the patient of that and schedule a consultation to discuss the results. When a severe abnormality is detected and the patient wishes to know the result, the ideal is to confer with the requesting physician on the best way to manage the case before informing the patient. When that is not possible, the radiologist should inform the patient that there is a problem that needs to be investigated and that a follow-up appointment with the requesting physician should be scheduled promptly^(5)^.

The code of medical ethics of the Brazilian Federal Medical Council states the following^(11)^: "It is forbidden for the physician . . . (article 34) to fail to inform a patient of the diagnosis and prognosis, as well as the risks and objectives of treatment, except when doing so could cause the patient harm, in which case the legal representative of the patient should be notified." That means that physicians must communicate the diagnosis, either directly to patients or to their caregiver or legal representative, even when a patient does not want to know the diagnosis. However, is the radiologist of today capable of doing this without causing harm to the patient, while also conforming to our code of medical ethics?

## CONCLUSION

The preparation of radiologists in effective physician-patient communication, albeit essential for long-term improvements in the specialty, has been neglected in medical residency programs. The written report is often insufficient to communicate a diagnosis, especially when it is unfavorable, as is often the case at a cancer hospital. The field of radiology is increasingly in need of better physician-patient communication, and there is no doubt that it is time for this to change.
